# Essential Contribution of CD4^+^ T Cells to Antigen-Induced Nasal Hyperresponsiveness in Experimental Allergic Rhinitis

**DOI:** 10.1371/journal.pone.0146686

**Published:** 2016-01-11

**Authors:** Tomoe Nishimura, Osamu Kaminuma, Mayumi Saeki, Noriko Kitamura, Kunie Matsuoka, Hiromichi Yonekawa, Akio Mori, Takachika Hiroi

**Affiliations:** 1 Allergy and Immunology Project, Tokyo Metropolitan Institute of Medical Science, Setagaya-ku, Tokyo, Japan; 2 Mammalian Genetics Project, Tokyo Metropolitan Institute of Medical Science, Setagaya-ku, Tokyo, Japan; 3 Basic Research Center, Tokyo Metropolitan Institute of Medical Science, Setagaya-ku, Tokyo, Japan; 4 Clinical Research Center for Allergy and Rheumatology, National Hospital Organization, Sagamihara National Hospital, Sagamihara, Kanagawa, Japan; Institute for Virus Research, Laboratory of Infection and Prevention, JAPAN

## Abstract

Nasal hyperresponsiveness (NHR) is a characteristic feature of allergic rhinitis (AR); however, the pathogenesis of NHR is not fully understood. In this study, during the establishment of an experimental AR model using ovalbumin-immunized and -challenged mice, augmentation of the sneezing reaction in response to nonspecific proteins as well as a chemical stimulant was detected. Whether NHR is independent of mast cells and eosinophils was determined by using mast cell- and eosinophil-deficient mice. NHR was suppressed by treatment with anti-CD4 antibody, suggesting the pivotal contribution of CD4^+^ T cells. Furthermore, antigen challenge to mice to which *in vitro*-differentiated Th1, Th2, and Th17 cells but not naïve CD4^+^ T cells had been adoptively transferred led to the development of equivalent NHR. Since antigen-specific IgE and IgG were not produced in these mice and since antigen-specific IgE-transgenic mice did not develop NHR even upon antigen challenge, humoral immunity would be dispensable for NHR. CD4^+^ T cells play a crucial role in the pathogenesis of AR via induction of NHR, independent of IgE-, mast cell-, and eosinophil-mediated responses.

## Introduction

Patients with allergic rhinitis (AR) develop several nasal symptoms including sneezing, rhinorrhea, and nasal congestion upon provocation with sensitized antigens. It is commonly considered that these symptoms are mediated by histamine and other chemical mediators derived from mast cells; therefore, IgE-mediated degranulation of these cells seems to have a crucial contribution to the induction of nasal responses [1,2]. AR is also characterized by submucosal inflammation associated with massive accumulation of inflammatory cells including eosinophils and T cells, like other allergic diseases [1,3].

Sneezing is a physiological response evoked by stimulation of the nasal mucosa by physical and chemical irritants. It is also known that the sneezing response to non-antigenic stimuli is augmented in AR patients. Threshold histamine doses for inducing this response are significantly lower in AR patients than in healthy controls and are inversely correlated with the severity of nasal symptoms [4–6]. In the case of seasonal nasal allergies such as pollinosis, the extrinsic histamine-induced sneezing response is enhanced in the pollen season but not during the off-season, and a positive correlation is seen with increase in the amount of nasal secretion [7].

The development of allergic inflammation in the nasal submucosa is related to nasal hyperresponsiveness (NHR); however, the mechanisms underlying enhancement of the sneezing response in patients with AR is still unclear. Histamine reactivity increases in AR patients on provocation with the corresponding antigen and the extent of increase is correlated with the number of eosinophils and neutrophils in nasal lavage fluid (NALF) [8–10]. Decrease in nasal eosinophil infiltration on beclomethasone treatment is also correlated with reduction of histamine-induced sneezing and *N*-alpha-tosyl L-arginine methyl ester esterase activity [9]. However, the contribution of these cell types to NHR has not been directly indicated.

Although a series of nasal symptoms is induced via the IgE/mast cell-dependent pathway, a crucial contribution of CD4^+^ T cells to the development of allergic inflammation in nasal submucosa has also been demonstrated. Increases in activated CD4^+^ T cells as well as T cell cytokines are detectable in the allergic nasal tissues and NALF of AR patients [11–13]. These responses are further increased by antigen provocation [14,15] and are inhibited by treatment with steroids and allergen specific immunotherapy, in association with the alleviation of nasal symptoms [11–13,16–19].

However, until date, there is not much evidence indicating the contribution of CD4^+^ T cells to the pathogenesis of AR, especially the development of NHR, despite numerous investigations that indicated a close relationship between CD4^+^ T cells and bronchial hyperresponsiveness (BHR) in asthmatic patients [20–24]. Therefore, in our present study, the role of CD4^+^ T cells in antigen-induced NHR was investigated. During the establishment of a murine model of AR, we found that the sneezing response evoked by nonspecific stimuli was enhanced in antigen-immunized and -challenged mice. By employing a variety of animals and materials including mast cell- and eosinophil-deficient mice, antigen-specific IgE-transgenic (Tg) mice, CD4^+^ cell-depleting antibody (Ab), and mice to which antigen-specific T cells had been adoptively transferred, an essential role of CD4^+^ T cells in the development of NHR was elucidated.

## Materials and Methods

### Animals

Six-week-old female BALB/c mice were purchased from Japan SLC (Shizuoka, Japan). DO11.10/RAG-2^-/-^ mice were generated and maintained for antigen-specific T cell preparation as described previously [22]. Mast cell-deficient (W/W^v^), eosinophil-deficient (ΔdblGATA), and anti-ovalbumin (OVA) IgE-Tg mice were introduced and maintained as previously reported [23–25]. The experimental protocols were approved (nos. 11–073, 12–36, 13057, 14027, and 15035) by the Animal Use and Care Committee of Tokyo Metropolitan Institute of Medical Science.

### *In vitro* polarization of T cells

Antigen-specific Th1, Th2, and Th17 cells were prepared as described previously [22,26]. Briefly, OVA-specific naïve CD4^+^ T cells were isolated from splenocytes of DO11.10/RAG-2^-/-^ mice by positive selection using CD4 microbeads and a magnetic cell sorting system (Miltenyi, Biotec BmbH, Bergisch Gladbach, Germany). Cells were cultured with X-ray-irradiated splenocytes in DMEM-F12/HAM medium (Sigma-Aldrich, MO, USA) supplemented with 10% fetal bovine serum. At the start of culture, 0.3 μM synthetic OVA323-339 peptide and 10 U/ml recombinant IL-2 (Shionogi, Osaka, Japan) were added. For the development of each subset, appropriate cytokines and anti-cytokine Abs were also added as described previously [22,27]. Seven days after the stimulation, cells were harvested and used for adoptive transfer. The polarization of T cell subsets was confirmed by flow cytometry with intracellular cytokine staining after stimulation with phorbol ester plus Ca^2+^ ionophore as described previously [22].

### Antigen immunization, cell transfer and challenge

**Fig 1:**
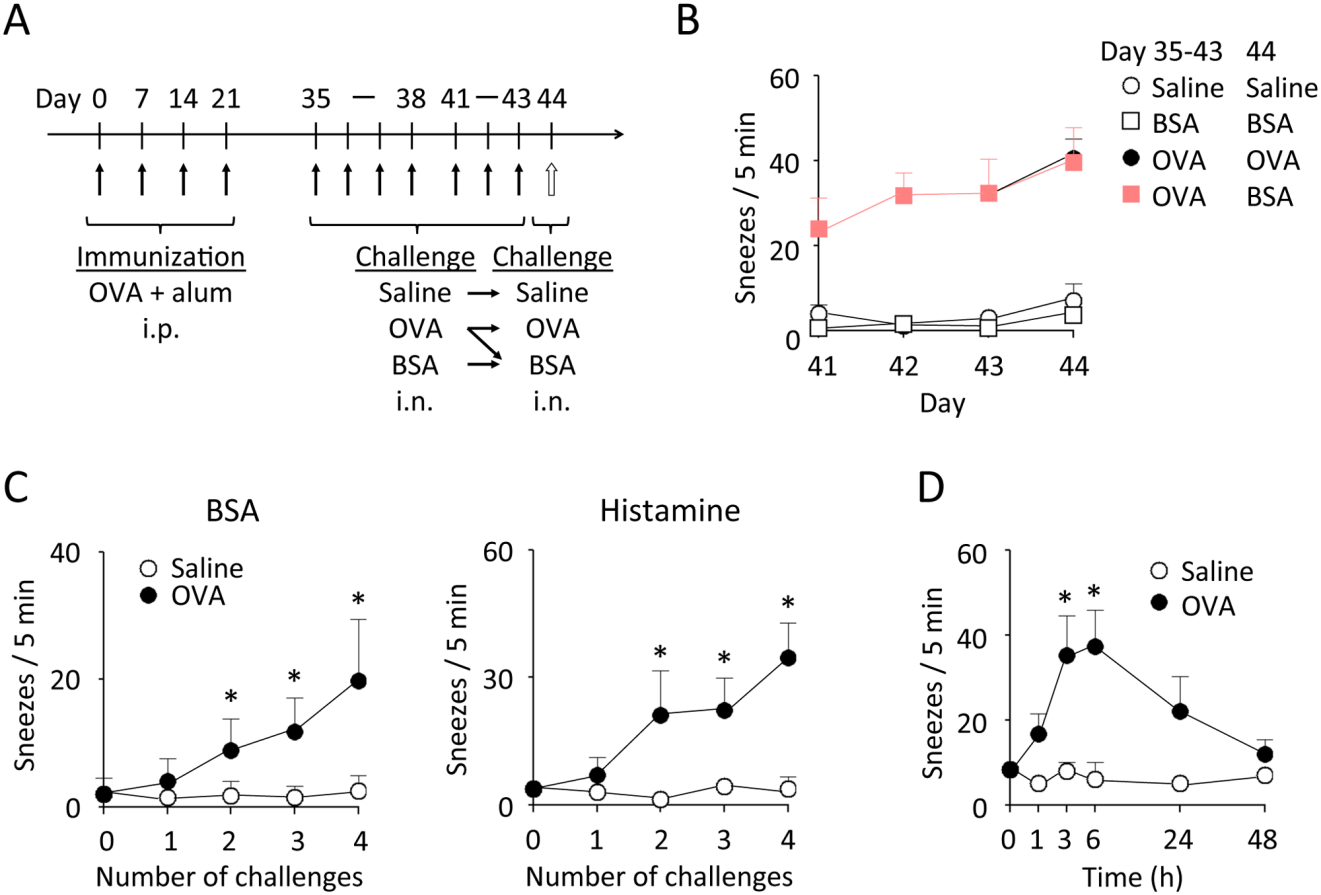
Antigen-induced NHR in immunized mice. Mice were immunized with 4-time i.p. injection of OVA plus alum. Two weeks after the last immunization, mice were challenged once a day with daily i.n. injection of OVA or BSA solution, or of saline on days 35–38 and 41–43. Then, these mice were challenged with OVA, BSA, or saline on day 44 (A). On days 41–44, the number of sneezes was counted for 5 min just after i.n. administration of OVA, BSA, or saline (B). The BSA- and histamine-evoked sneezing response was evaluated at 6 h after 0 (day 34)- to 4 (day 38)-time challenge with OVA or saline (C). Time course of histamine-evoked sneezing response after 4-time challenge on days 35–38 with OVA or saline was evaluated (D). Data are expressed as mean ± SEM for 4–10 animals. *p < 0.05, compared with saline-challenged control mice (Mann-Whitney *U* test).

Mice were immunized 4 times by weekly intraperitoneal (i.p.) injection of 20 μg OVA (Sigma-Aldrich) emulsified in 2.25 mg alum (Inject Alum; Thermo Scientific, IL, USA). Two weeks after the last immunization, the mice were challenged once a day with intranasal (i.n.) injection of 5 μl OVA, bovine serum albumin (BSA) (Sigma-Aldrich), or casein (Sigma-Aldrich) solution (30 mg/ml in saline) without anesthesia for 4 consecutive days. For the initial examination and experiments with W/W^v^ and ΔdblGATA mice, the same challenge was repeated after a 3-day interval (Fig 1A). In some experiments, 50 mg/kg anti-CD4 monoclonal Ab (mAb) (GK1.5, eBioscience) was administered intravenously (i.v.) twice, that is, at 9 and 6 days before the last antigen challenge. The resulting depletion of CD4^+^ cells was confirmed by flow cytometry for splenocytes stained with anti-CD3-PECy7 (BioLegend, CA, USA) and anti-CD4-APC eFluor780 (eBioscience, CA, USA) Abs.

In the T cell transfer model, polarized Th1, Th2, and Th17 cells as well as naïve CD4^+^ T cells (2 × 10^7^) were injected i.v. in each mouse. Twenty-four hours later, the mice were challenged by i.n. administration of OVA or saline once a day for 3 consecutive days. The accumulation of transferred Th2 cells in NALF and nasal associated lymphoid tissue (NALT) was evaluated by flow cytometry upon staining with anti-DO11.10-TCR-PE (BD Bioscience, CA, USA) and anti-CD4-APC eFluor780 Abs.

Serum levels of antigen-specific immunoglobulins in these mice were measured by ELISA using HRP-conjugated anti-mouse IgE mAb (Serotech, Oxford, UK) and goat anti-mouse IgG, IgG_1_, IgG_2a_, IgG_2b_, and IgG_3_ (Southern Biotech Associates, Birmingham, AL) Abs for detection, as described previously [28]. Data are presented as the optical density (O.D.) values measured at 450 nm.

### NHR, nasal lavage (NAL), and histological analyses

NHR was assessed by counting the number of sneezes for 5 min just after i.n. administration of 10 μl each of several proteins (30 mg/ml) and histamine (100 mM except for a dose-response study). NAL analysis was performed 6 h after the last antigen challenge. Inflammatory cells in the NALF were classified by means of morphological criteria as described previously [22,29]. Lateral nose sections (5 μm thick) were stained with hematoxylin and eosin and observed under optical microscopy. Subsequently the number of infiltrated eosinophils was determined and the epithelial damage was evaluated as described previously [30] by grading 0 for normal epithelium, 1 for cilia loss, 2 for eroded upper cell layer and intact basal cell layer, and 3 for eroded epithelium.

Total RNA was extracted from the nasal tissue. After reverse transcription using a random primer (Toyobo, Osaka, Japan) and SuperScript III reverse transcriptase (Thermo Fisher Scientific, Inc., Waltham, MA), quantitative real-time RT-PCR for IFN-γ, IL-4, IL-5, IL-13, IL-17, and eosinophil peroxidase (EPO) was performed using Assay-on-Demand^TM^ Gene Expression Products (TaqMan^®^ MGB probes, Thermo Fisher Scientific, Inc.) with a CFX96^TM^ Real-Time PCR Detection System (Bio-Rad, Hercules, CA) as described previously [31].

### Statistical analysis

The results have been presented as arithmetic mean ± SEM. Statistical analysis was performed using Mann-Whitney *U* test for comparison between two groups and Kruskal-Wallis test with Dunn’s multiple comparison test for three groups or more. *p* < 0.05 was considered to indicate statistical significance.

## Results

### Antigen-induced NHR in immunized mice

This experiment was performed in order to establish an AR model in mice. Intranasal OVA challenge to immunized BALB/c mice (Fig 1A) induced a sneezing response, and the extent of this response gradually increased following repeated antigen challenge (Fig 1B). This seemed to be antigen-specific since a nonspecific protein, BSA, failed to induce significant sneezing even with repeated challenge. However, challenge with BSA after 7-time OVA challenge evoked sneezes equivalent to the number induced by OVA (Fig 1B). The induction of a sneezing response by BSA was already observed after only 2 times OVA challenge, and its extent increased following repeated antigen challenge (Fig 1C). Furthermore, the antigen-induced increase in sneezing response was also detectable using histamine (Fig 1C) and casein (S1A Fig) as final stimulants. Intranasal administration of histamine could induce sneezing in a concentration-dependent manner even in saline-challenged mice; this response was significantly enhanced in antigen-challenged mice (S1B Fig). These findings suggest that NHR is induced in antigen-immunized mice upon antigen provocation. NHR was significantly detectable at 3 h after 4-time antigen challenge, peaked at 6 h, and disappeared by 48 h (Fig 1D).

**Fig 2:**
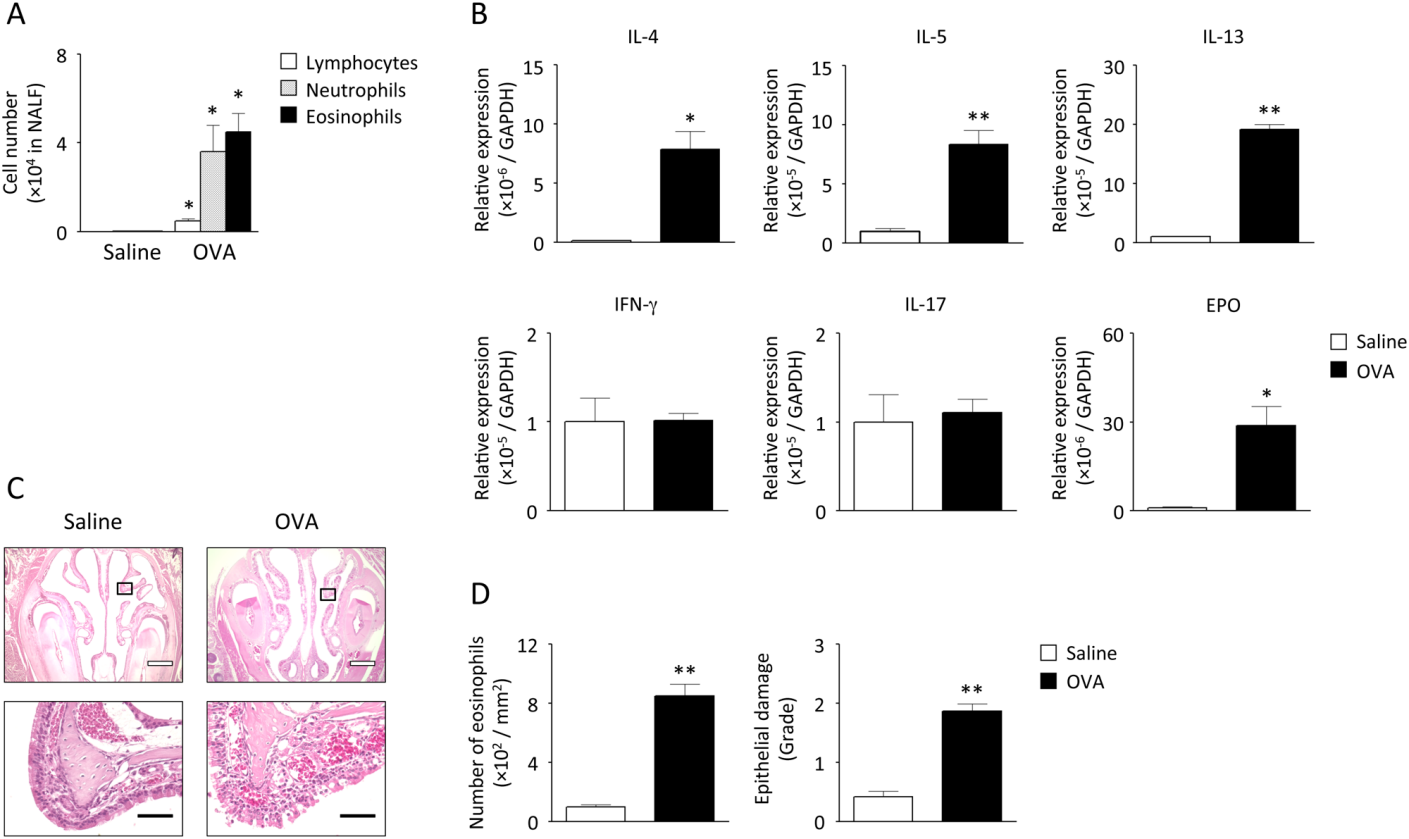
Antigen-induced nasal inflammation in immunized mice. Immunized mice were challenged 4 times with OVA or saline on days 35–38. Six hours after the last challenge, accumulation of lymphocytes, neutrophils, and eosinophils in NALF (A) and expression of IL-4, IL-5, IL-13, IFN-γ, IL-17, and EPO mRNA in the nasal tissue (B) were examined. Data are expressed as mean ± SEM for 4–9 animals. *p < 0.05, **p < 0.01, compared with saline-challenged control mice (Mann-Whitney *U* test). Lateral nose sections were stained with hematoxylin and eosin and observed under optical microscopy. Representative images from 3 independent experiments are shown in panel C. The lower panels are enlarged views of the squares in the upper panels. White and black bars indicate 500 and 50 μm, respectively. The number of infiltrated eosinophils and the grade of epithelial damage are evaluated from the histological images (D). Data are expressed as mean ± SEM. **p < 0.01, compared with saline-challenged control mice (Mann-Whitney *U* test).

Six hours after 4-time repeated antigen challenge, the numbers of lymphocytes, eosinophils, and neutrophils in the NALF significantly increased (Fig 2A). At that time point, mRNA expression of IL-4, IL-5, IL-13, and EPO significantly increased on OVA challenge, whereas the expression of IFN-γ and IL-17 was unchanged (Fig 2B). Disarrangement of epithelial layers as well as massive accumulation of inflammatory cells, including eosinophils was also observed in the nasal submucosa of OVA-challenged mice (Fig 2C and 2D).

### Contribution of mast cells, eosinophils and CD4^+^ T cells to NHR

**Fig 3:**
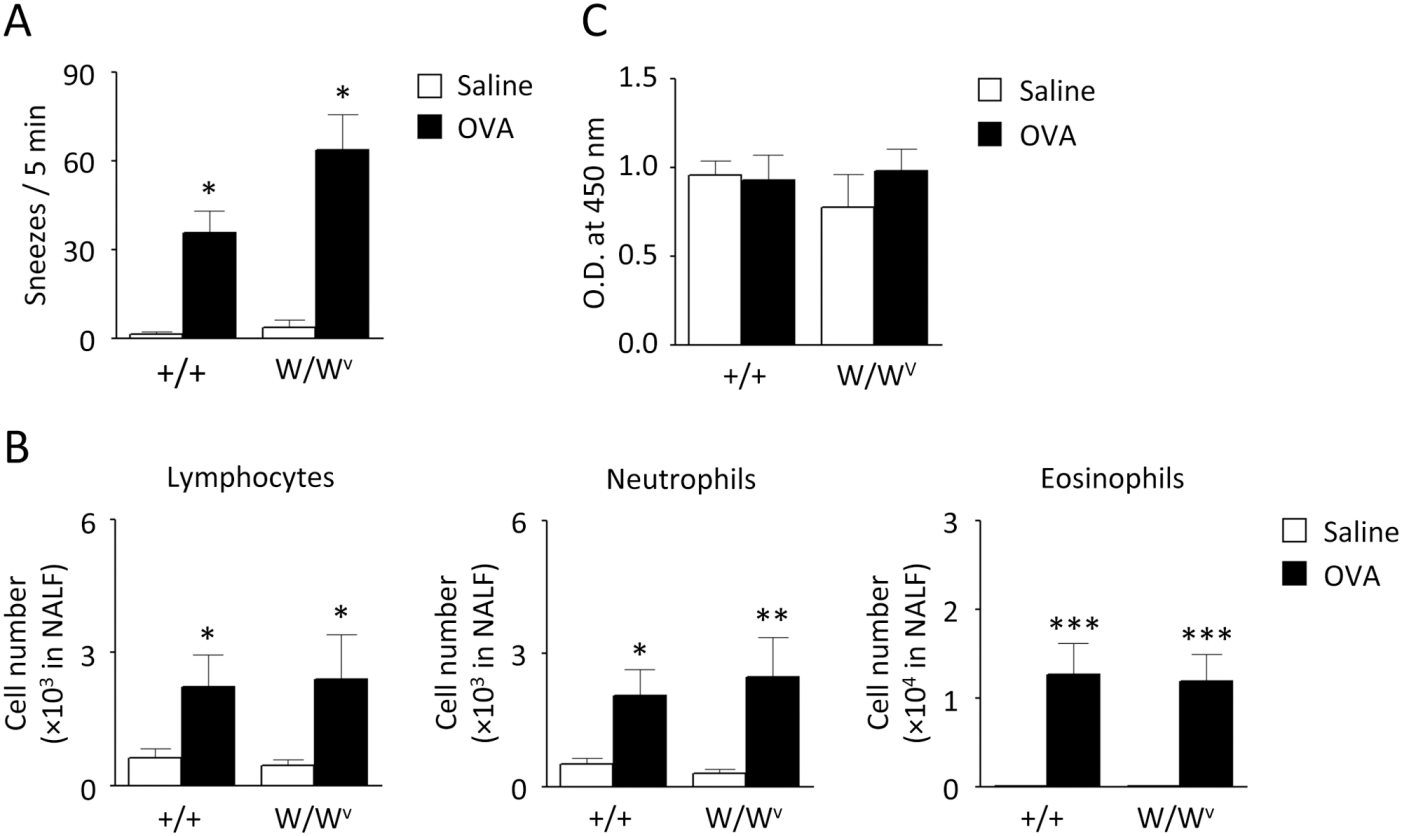
Antigen-induced NHR in mast cell-deficient mice. Immunized W/W^v^ and +/+ mice were challenged 7 times with OVA or saline, as shown in Fig 1. Six hours after the last challenge (day 43), the number of sneezes evoked by histamine (A), the accumulation of lymphocytes, neutrophils, and eosinophils in NALF (B), and the antigen-specific serum IgE levels were examined (C). Data are expressed as mean ± SEM for 4–9 animals. *p < 0.05, **p < 0.01, ***p < 0.001, compared with saline-challenged control mice (Mann-Whitney *U* test).

**Fig 4:**
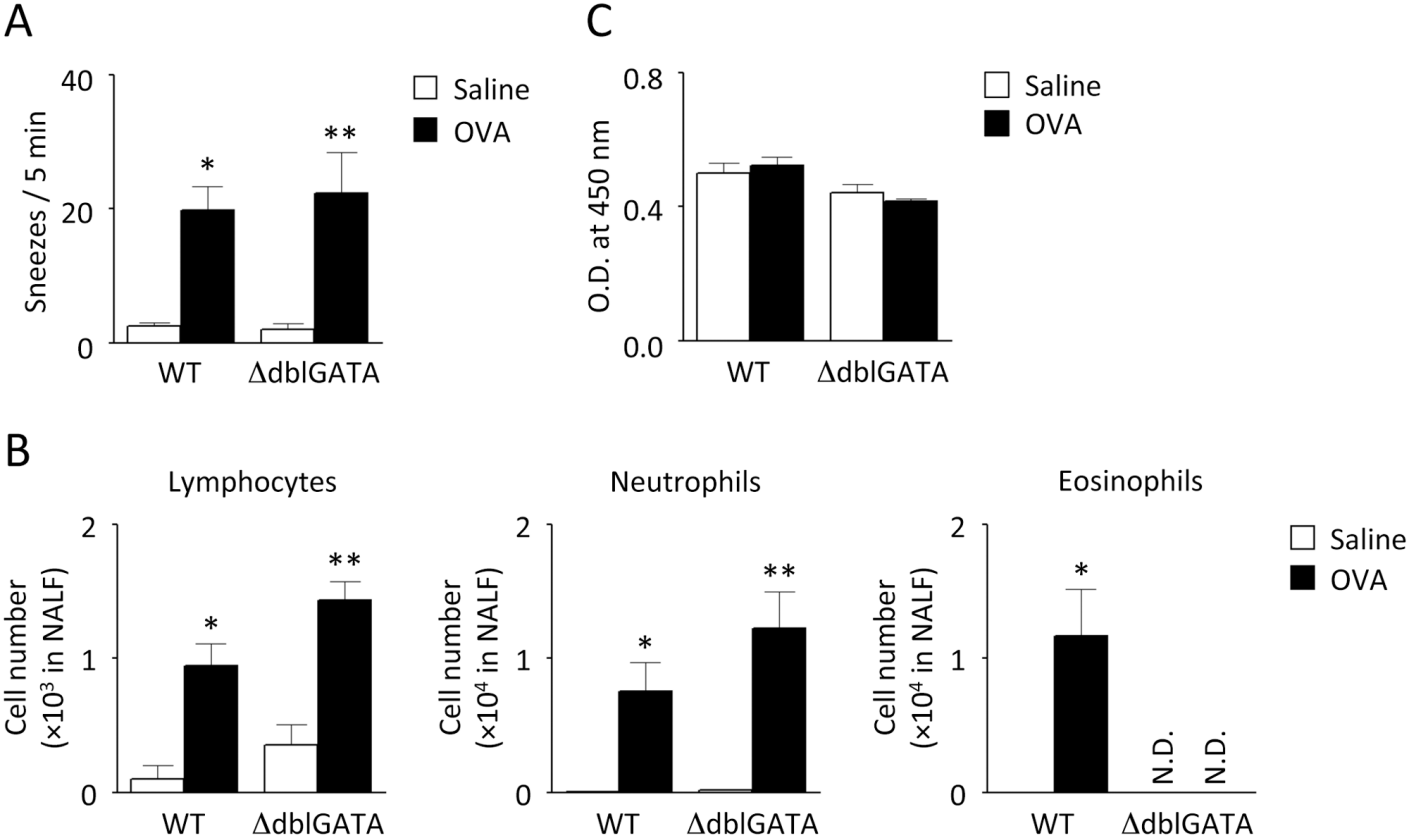
Antigen-induced NHR in eosinophil-deficient mice. Immunized ΔdblGATA and WT mice were challenged 7 times with OVA or saline, as shown in Fig 1. Six hours after the last challenge (day 43), the number of sneezes evoked by histamine (A), the accumulation of lymphocytes, neutrophils, and eosinophils in NALF (B), and the antigen-specific serum IgE levels were examined. Data are expressed as mean ± SEM for 4–8 animals. *p < 0.05, **p < 0.01, compared with saline-challenged control mice (Mann-Whitney *U* test). N.D.: not detectable.

To elucidate the mechanisms underlying NHR, the contributions of mast cells and eosinophils were examined by employing mice deficient in these cells. Antigen-induced increase in histamine-evoked sneezing response (Fig 3A), nasal infiltration of inflammatory cells (Fig 3B), and serum IgE response (Fig 3C) were similarly observed in W/W^v^ mice and their congenic littermates (+/+). Furthermore, the extent of antigen-induced NHR was equivalent in ΔdblGATA and wild-type (WT) mice (Fig 4A and S2 Fig) although nasal accumulation of eosinophils was completely abrogated in ΔdblGATA mice (Fig 4B). The numbers of lymphocytes and neutrophils recovered in the NALF of ΔdblGATA mice upon OVA challenge were not significantly different from those of WT mice (Fig 4B). Essentially the same antigen-specific IgE response was also observed in ΔdblGATA and WT mice (Fig 4C).

**Fig 5:**
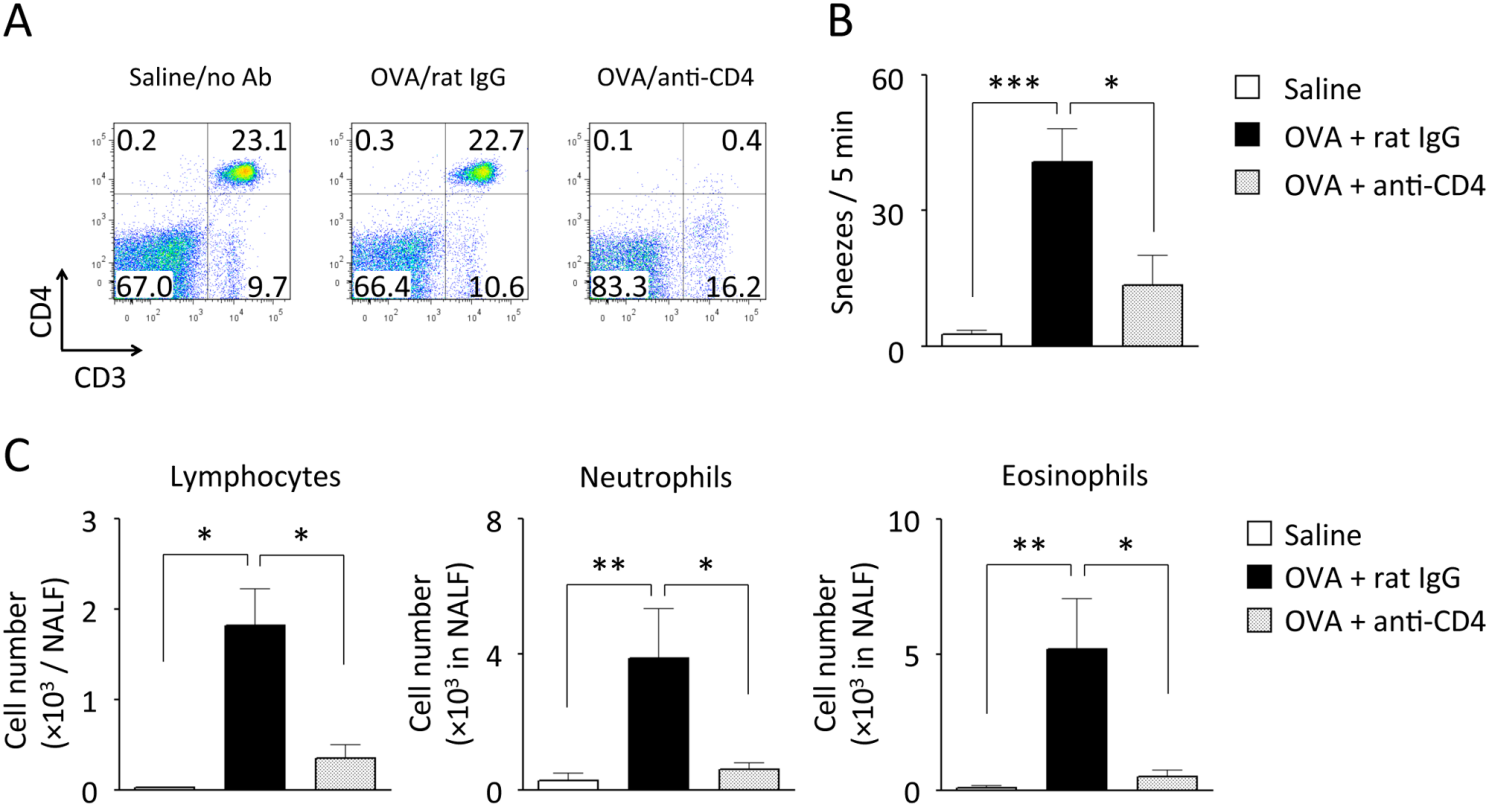
Effect of anti-CD4 mAb on antigen-induced NHR. Immunized mice were challenged 4 times with OVA or saline, as shown in Fig 2. Anti-CD4 mAb or control rat IgG was administered twice, that is, at 9 and 6 days before the last challenge. Six hours after the last challenge, the CD3^+^CD4^+^ population in the spleen was determined by flow cytometry (A). Representative data from 3 independent experiments are shown. The number of sneezes evoked by histamine (B) and the accumulation of lymphocytes, neutrophils, and eosinophils in NALF (C) were also examined. Data are expressed as mean ± SEM for 4–6 animals. *p < 0.05, **p < 0.01, ***p < 0.01, compared with OVA-challenged control mice (Dunn’s test).

A pivotal role of CD4^+^ T cells in antigen-induced NHR was elucidated by the CD4 depletion study. Administration of an anti-CD4 mAb to immunized mice depleted peripheral CD4^+^ T cells almost completely (Fig 5A). Consequently, antigen-induced increase in the histamine-evoked sneezing response was significantly suppressed (Fig 5B). Nasal infiltration of eosinophils, neutrophils and lymphocytes was also significantly diminished (Fig 5C). These findings suggest that CD4^+^ T cells, but not mast cells or eosinophils, are required for the induction of NHR.

### Antigen-specific T cells confer NHR

**Fig 6:**
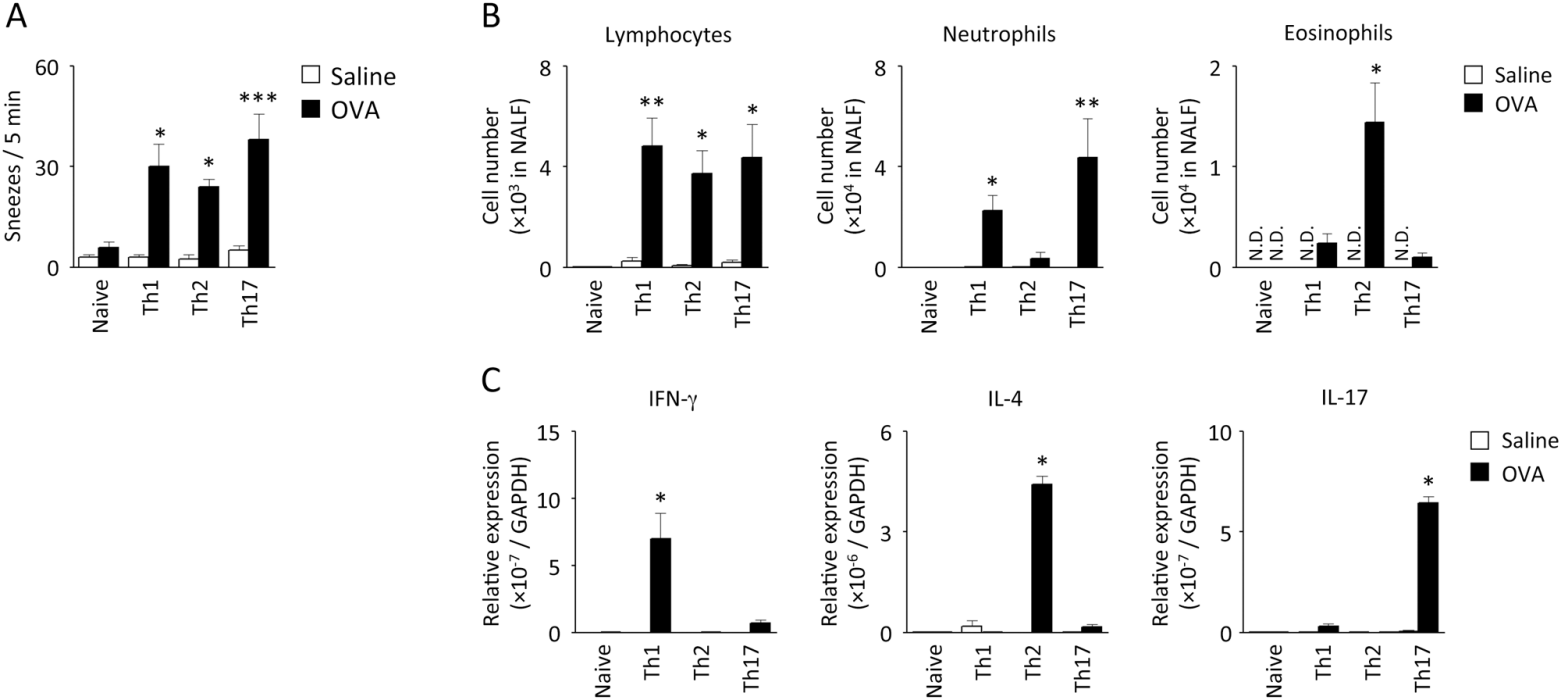
Antigen-induced NHR in T cell-transferred mice. Twenty-four hours after transfer of Th1, Th2, or Th17-polarized cells, mice were challenged 3 times with OVA or saline. Six hours after the last challenge, the number of sneezes evoked by histamine (A), the accumulation of lymphocytes, neutrophils, and eosinophils in NALF (B), and the IFN-γ, IL-4, and IL-17 mRNA expression in the nasal tissue (C) were examined. Data are expressed as mean ± SEM for 4–8 animals. *p < 0.05, **p < 0.01, ***p < 0.001, compared with naïve T cell-transferred and OVA-challenged mice (Dunn’s test). N.D.: not detectable.

After determining the essential role of CD4^+^ T cells in NHR, we investigated whether T cells by themselves could induce NHR. Antigen-specific Th1, Th2, and Th17 cells were established by *in vitro* stimulation culture. After the assessment of adequate differentiation of each subset by intracellular cytokine staining [22], T cells were adoptively transferred to normal mice. Upon antigen provocation, significant increase in histamine (Fig 6A)- and BSA (S3A Fig)-induced sneezing response was observed in mice to which Th1, Th2, and Th17 cells had been transferred. In the NAL examination, eosinophil-dominant cellular infiltration was observed in the case of Th2 transfer, whereas neutrophil-rich nasal inflammation was induced by Th1 and Th17 (Fig 6B). Furthermore, antigen-induced accumulation of transferred T cells (DO11.10 TCR^+^CD4^+^ cells) in NALF and NALT was confirmed for Th2 transfer (S3B Fig). IFN-γ, IL-4, and IL-17A mRNA expression was preferentially induced in the case of Th1, Th2, and Th17 transfer, respectively, by antigen challenge (Fig 6C), suggesting that these subsets exhibited their representative phenotypes *in vivo*. Antigen challenge to naïve CD4^+^ T cell-transferred mice failed to induce significant NHR, nasal accumulation of inflammatory cells, and cytokine expression (Fig 6).

### Role of IgE in NHR

**Fig 7:**
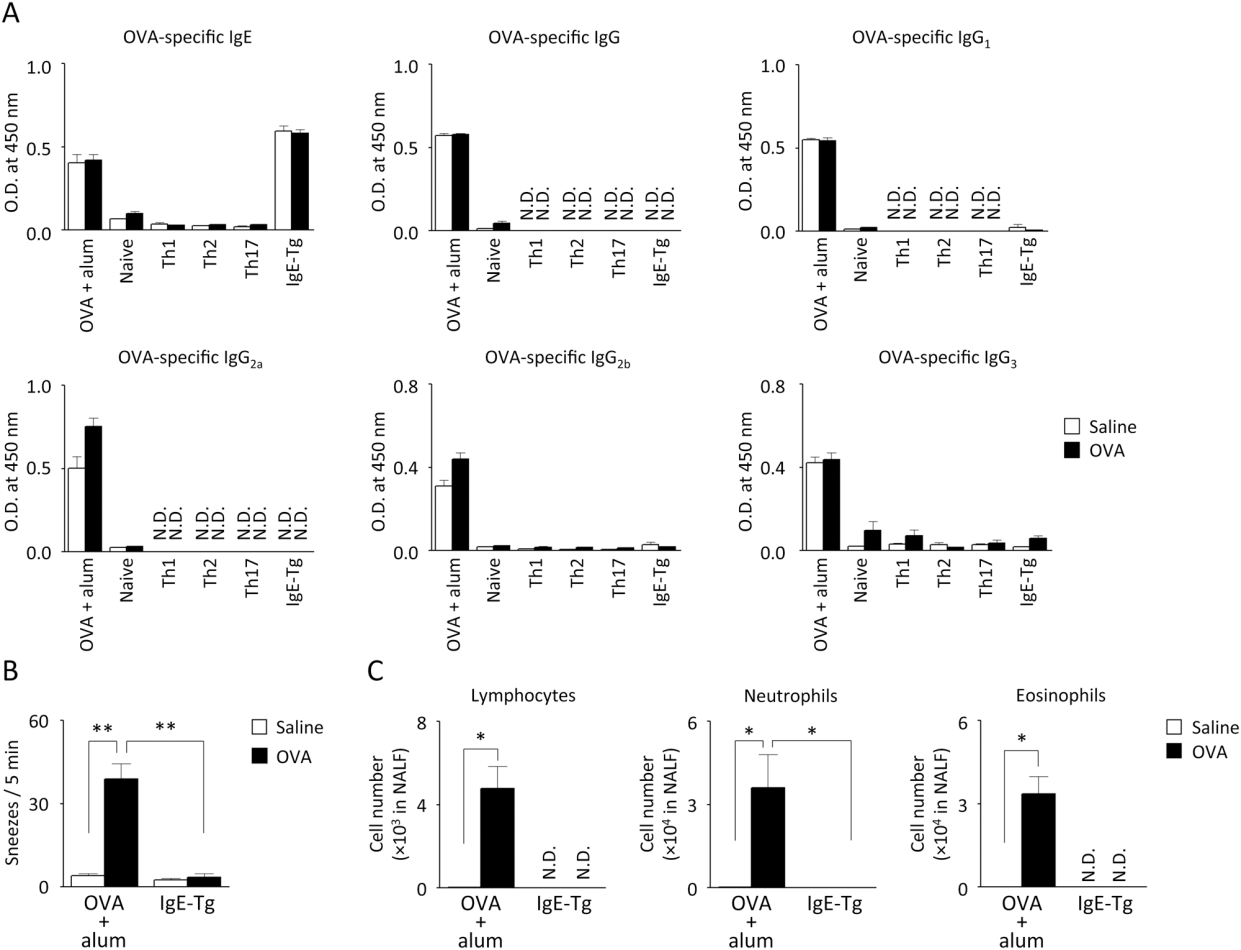
Defect in antigen-induced NHR in antigen-specific IgE-Tg mice. IgE-Tg mice as well as antigen-immunized mice and T cell-transferred mice were challenged 3–4 times with OVA or saline, as shown in Figs 2 and 6. Six hours after the 3rd challenge, antigen-specific serum IgE, IgG, IgG_1_, IgG_2a_, IgG_2b_, and IgG_3_ were determined (A). The number of sneezes evoked by histamine (B) and accumulation of lymphocytes, neutrophils, and eosinophils in NALF (C) of IgE-Tg mice were examined 6 h after the 4th challenge. Data are expressed as mean ± SEM for 4–8 animals. *p < 0.05, **p < 0.01 (Dunn’s test). N.D.: not detectable.

Although significant contribution of mast cells to NHR was improbable, antigen-specific IgE could still play some roles in allergic responses via the activation of basophils. Therefore, the contribution of IgE to the development of NHR was investigated by employing anti-OVA IgE-Tg mice. In contrast to mice to which T cells had been transferred and which did not produce significant amounts of OVA-specific IgE as well as IgG and its subclasses, a large amount of antigen-specific IgE was detected in the sera of IgE-Tg mice (Fig 7A). The serum IgE levels in IgE-Tg mice were equivalent to those in antigen-immunized wild-type mice, whereas antigen-specifc IgG and its subclasses were almost undetectable in IgE-Tg mice. Antigen-specific IgE and IgG were also not detectable in unimmunized naive mice (data not shown). An increase in the histamine—evoked sneezing response was not observed in IgE-Tg mice even upon antigen challenge (Fig 7B), and there was almost no recovery of inflammatory cells in the NALF (Fig 7C).

## Discussion

Our present study clearly demonstrated that the development of NHR in antigen-immunized and -challenged mice was dependent on CD4^+^ T cells but not on mast cells and eosinophils. The existence of differentiated antigen-specific T cells was sufficient, whereas humoral immunity was dispensable, for the induction of NHR.

Since IgE and mast cells are crucially involved in the pathogenesis of AR [1,2,9,32], it is intriguing that they played an inconsequential role in the development of NHR. Despite recent advance in the knowledge of the pivotal role of basophils in some allergic situations [33,34], IgE-dependent activation of basophils also does not seem to cause NHR, at least under our experimental conditions.

The symptoms and pathological features of AR could be reproduced, at least in part, in its murine models. For example, nasal antigen challenge to immunized mice evoked sneezing and nasal rubbing, as well as infiltration of inflammatory cells into the nasal submucosa in these mice [35–38]. In addition, several murine models exhibiting antigen-induced increase in sneezing response have been developed [39,40], although the mechanistic analysis has been poorly performed in these studies. By means of serial experiments employing an antigen-immunization model with CD4 depletion and an antigen-specific T cell-transfer model, we clarified an indispensable and sufficient contribution of CD4^+^ T cells to the development of antigen-induced NHR. Since patients with NHR are likely to suffer from severe and more often AR symptoms than those without NHR, CD4^+^ T cell could directly contribute to the exacerbation of AR. This was consistent with the clinical observation that beclomethasone, which targets T cell activation, inhibits antigen-induced NHR as well as nasal symptoms and inflammation in AR patients [9]. Even in a murine model, the suppression of antigen-induced nasal inflammation is achieved by using tacrolimus, another inhibitor of T cell activation [41].

Previous studies by Yoshimoto’s group reported an enhanced sneezing response in immunized mice after repeated ragweed antigen challenge [38,42]. According to our present findings, this response was due, at least in part, to NHR, although Yoshimoto’s group did not mention it in their studies. However, contradictory to our findings, they reported that the augmentation of sneezing response was diminished by employing W/W^v^ and FcεRI^-/-^ mice. They concluded that IL-33-mediated increase in histamine release from the mast cells and basophils was important for antigen-induced augmentation of sneezing response. The appropriate reason for such discrepancy is not known, however, it is possibly related to the difference in the antigens used. It has recently been demonstrated that ragweed antigen exhibits cysteine protease activity and directly activate human basophils [43]. Some active substance that directly stimulates mast cell degranulation might also be present in the crude ragweed pollen extract used by Yoshimoto’s group. Base on this, it is possible that mast cells as well as eosinophils still contribute to NHR when the sneezing response is evoked by cell type-specific nasal stimulants.

The mechanisms of NHR have also been investigated in animal models, employing nasal obstruction and secretion as outcomes. Thus, treatment with a muscarinic M1/M3 antagonist attenuates antigen-induced mucus hypersecretion in rats [35]. Pharmacological intervention of neurokinin 2 and bradikinin 1/2 receptors inhibits antigen-induced increase in the nasal occlusion response in guinea pigs [44,45]. In this regard, T cells could produce several neuropeptides such as substance P [46,47]. Preprotachykinin-A-encoding substance P and tackykinins are expressed in CD3^+^ T cells and are upregulated by stimulation with phytohemagglutinin [48]. Although the physiological process of nasal obstruction and secretion is different from that of sneezing, these neural transmitters might be involved in the CD4^+^ T cell-mediated enhancement of sneezing response in AR. Further clinical and animal investigations on the role of CD4^+^ T cell-derived neurotransmitters along with monitoring nasal obstruction, secretion, and sneezing response is important to understand the detailed pathogenesis of NHR.

It is also interesting that the present findings quite resemble our previous observations obtained in the lower airways of murine asthma models. Thus, antigen-induced BHR is abrogated by the depletion of CD4^+^ T cells, although it develops similarly in W/W^v^ and +/+ mice [24]. Adoptive transfer of antigen-specific Th1, Th2, and Th17 cells confers BHR to normal mice upon antigen challenge [22]. A close relationship between CD4^+^ T cells and BHR was also confirmed in several clinical studies on bronchial asthma [20,21]. Although the organ structure and physiological output are quite different between the upper and lower airways, and although BHR was not developed in the current experimental conditions [37], essentially the same mechanisms might be involved in T cell-dependent NHR and BHR.

The dispensable role of eosinophils in NHR is another remarkable finding in this study because their importance in allergic diseases has been reported. In particular, BHR was considered to be mediated by eosinophils accumulated in the bronchial submucosa of patients with bronchial asthma [49]. However, recent conflicting findings observed in eosinophil-deficient mice [50] and in clinical studies targeting eosinophil-activating cytokines [51] have led to controversies regarding the role of this cell type in BHR. Furthermore, in the ragweed-induced AR model established by Yoshimoto’s group, reduced sneezing response but equivalent nasal eosinophil infiltration was observed in FcεRI^-/-^ mice [42]. Although the contribution of eosinophils in AR is considered to be lesser than that in asthma, our present results might be helpful for understanding the exact role of eosinophils in the pathogenesis of allergic diseases.

Since neutrophil-derived elastase and cyclooxygenase products could cause BHR [52,53], neutrophils might participate to some extent in NHR in mice to which Th1 and Th17 cells are transferred. However, the contribution of this cell type to Th2-mediated NHR seems to be negligible, suggesting that CD4^+^ T cells could induce NHR without assistance of neutrophils as well as eosinophils.

Taking these findings together, the existence of a molecule that is expressed or derived by CD4^+^ T cells but not eosinophils, neutrophils, or mast cells and induces NHR is suggested. Although T cell subsets are characterized by their specific cytokine production profiles, they might commonly produce several cytokines or neurotransmitters. Finding mediators similarly produced by Th1, Th2, and Th17 cells but not by naïve CD4^+^ T cells is the next step toward the identification of the cascade from CD4^+^ T cells to the development of NHR.

## Supporting Information

S1 Fig Sneezing response evoked by specific and nonspecific stimulants in OVA-immunized and -challenged mice. The number of sneezes evoked by OVA, BSA, or casein (A), or several concentrations of histamine (B) in OVA-immunized and saline- or OVA-challenged mice (N = 4–8).
(TIF)

S2 Fig Antigen-induced NHR in eosinophil-deficient mice. The number of sneezes evoked by BSA in OVA-immunized and saline- or OVA-challenged ΔdblGATA and WT mice (N = 4–10).
(TIF)

S3 Fig Antigen-induced NHR in T cell-transferred mice. The number of sneezes evoked by BSA in saline- or OVA-challenged Naïve, Th1, Th2, or Th17 cell-transferred mice (N = 4–8) (A). The DO11.10-TCR^+^CD4^+^ population in the NALF and NALT in saline- or OVA-challenged Th2-transferred mice (B).
(TIF)

## References

[1] Baraniuk JN (2001) Mechanisms of allergic rhinitis. Curr Allergy Asthma Rep 1: 207–217. 11892038 10.1007/s11882-001-0007-5

[2] Parikh SA, Cho SH, Oh CK (2003) Preformed enzymes in mast cell granules and their potential role in allergic rhinitis. Curr Allergy Asthma Rep 3: 266–272. 12662477 10.1007/s11882-003-0049-y

[3] Sin B, Togias A (2011) Pathophysiology of allergic and nonallergic rhinitis. Proc Am, Thorac Soc 8: 106–114. 21364228 10.1513/pats.201008-057RN

[4] Kanthawatana S, Maturim W, Fooanant S, Manorot M, Trakultivakorn M (1997) Evaluation of threshold criteria for the nasal histamine challenge test in perennial allergic rhinitis. Asian Pac J Allergy Immunol 15: 65–69. 9346268

[5] Sanico AM, Koliatsos VE, Stanisz AM, Bienenstock J, Togias A (1999) Neural hyperresponsiveness and nerve growth factor in allergic rhinitis. Int Arch Allergy Immunol 118: 154–158. 10224365 10.1159/000024054

[6] Gerth Van Wijk R, Dieges PH (1987) Comparison of nasal responsiveness to histamine, methacholine and phentolamine in allergic rhinitis patients and controls. Clin Allergy 17: 563–570. 3325190 10.1111/j.1365-2222.1987.tb02052.x

[7] Konno A, Togawa K, Nishihira S (1981) Seasonal variation of sensitivity of nasal mucosa in pollinosis. Arch Otorhinolaryngol 232: 253–261. 7305729 10.1007/BF00457450

[8] Walden SM, Proud D, Lichtenstein LM, Kagey-Sobotka A, Naclerio RM (1991) Antigen-provoked increase in histamine reactivity. Observations on mechanisms. Am Rev Respir Dis 144: 642–648. 1892305 10.1164/ajrccm/144.3_Pt_1.642

[9] Baroody FM, Cruz AA, Lichtenstein LM, Kagey-Sobotka A, Proud D, Naclerio RM (1992) Intranasal beclomethasone inhibits antigen-induced nasal hyperresponsiveness to histamine. J Allergy Clin Immunol 90: 373–376. 1527319 10.1016/s0091-6749(05)80017-x

[10] de Graaf-in t Veld C, Garrelds IM, Koenders S, Gerth van Wijk R (1996) Relationship between nasal hyperreactivity, mediators and eosinophils in patients with perennial allergic rhinitis and controls. Clin Exp Allergy 26: 903–908. 8877155

[11] Bradding P, Feather IH, Wilson S, Holgate ST, Howarth PH (1995) Cytokine immunoreactivity in seasonal rhinitis: regulation by a topical corticosteroid. Am J Respir Crit Care Med 151: 1900–1906. 7767538 10.1164/ajrccm.151.6.7767538

[12] Masuyama K, Till SJ, Jacobson MR, Kamil A, Cameron L, Juliusson S, et al (1998) Nasal eosinophilia and IL-5 mRNA expression in seasonal allergic rhinitis induced by natural allergen exposure: effect of topical corticosteroids. J Allergy Clin Immunol 102: 610–617. 9802369 10.1016/s0091-6749(98)70277-5

[13] Ghaffar O, Laberge S, Jacobson MR, Lowhagen O, Rak S, Durham SR, et al (1997) IL-13 mRNA and immunoreactivity in allergen-induced rhinitis: comparison with IL-4 expression and modulation by topical glucocorticoid therapy. Am J Respir Cell Mol Biol 17: 17–24. 9224205 10.1165/ajrcmb.17.1.2696

[14] Cameron L, Hamid Q, Wright E, Nakamura Y, Christodoulopoulos P, Muro S, et al (2000) Local synthesis of epsilon germline gene transcripts, IL-4, and IL-13 in allergic nasal mucosa after ex vivo allergen exposure. J Allergy Clin Immunol 106: 46–52. 10887304 10.1067/mai.2000.107398

[15] Nouri-Aria KT, O'Brien F, Noble W, Jabcobson MR, Rajakulasingam K, Durham SR (2000) Cytokine expression during allergen-induced late nasal responses: IL-4 and IL-5 mRNA is expressed early (at 6 h) predominantly by eosinophils. Clin Exp Allergy 30: 1709–1716. 11122208 10.1046/j.1365-2222.2000.00998.x

[16] Durham SR, Ying S, Varney VA, Jacobson MR, Sudderick RM, Mackay IS, et al (1996) Grass pollen immunotherapy inhibits allergen-induced infiltration of CD4+ T lymphocytes and eosinophils in the nasal mucosa and increases the number of cells expressing messenger RNA for interferon-gamma. J Allergy Clin Immunol 97: 1356–1365. 8648033 10.1016/s0091-6749(96)70205-1

[17] Masuyama K, Jacobson MR, Rak S, Meng Q, Sudderick RM, Kay AB, et al (1994) Topical glucocorticosteroid (fluticasone propionate) inhibits cells expressing cytokine mRNA for interleukin-4 in the nasal mucosa in allergen-induced rhinitis. Immunology 82: 192–199. 7927488 PMC1414820

[18] Varney VA, Hamid QA, Gaga M, Ying S, Jacobson M, Frew AJ, et al (1993) Influence of grass pollen immunotherapy on cellular infiltration and cytokine mRNA expression during allergen-induced late-phase cutaneous responses. J Clin Invest 92: 644–651. 8349803 10.1172/JCI116633 PMC294897

[19] Durham SR, Kay AB, Hamid Q (1995) Changes in allergic inflammation associated with successful immunotherapy. Int Arch Allergy Immunol 107: 282–284. 7613151 10.1159/000237003

[20] Wills-Karp M (1999) Immunologic basis of antigen-induced airway hyperresponsiveness. Annu Rev Immunol 17: 255–281. 10358759 10.1146/annurev.immunol.17.1.255

[21] Gelfand EW (1998) Essential role of T lymphocytes in the development of allergen-driven airway hyperresponsiveness. Allergy Asthma Proc 19: 365–369. 9876776 10.2500/108854198778612744

[22] Kaminuma O, Ohtomo T, Mori A, Nagakubo D, Hieshima K, Ohmachi Y, et al (2012) Selective down-regulation of Th2 cell-mediated airway inflammation in mice by pharmacological intervention of CCR4. Clin Exp Allergy 42: 315–325. 10.1111/j.1365-2222.2011.03847.x 22092376

[23] Ohtomo T, Kaminuma O, Yamada J, Kitamura N, Abe A, Kobayashi N, et al (2010) Eosinophils are required for the induction of bronchial hyperresponsiveness in a Th transfer model of BALB/c background. Int Arch Allergy Immunol 152 Suppl 1: 79–82. 10.1159/000312130 20523068

[24] Ogawa K, Kaminuma O, Kikkawa H, Nakata A, Asahina M, Egan RW, et al (2002) Transient contribution of mast cells to pulmonary eosinophilia but not to hyper-responsiveness. Clin Exp Allergy 32: 140–148. 12002731 10.1046/j.0022-0477.2001.01248.x

[25] Nishimura T, Saeki M, Kaminuma O, Matsuoka K, Yonekawa H, Mori A, et al (2013) Existence of antigen-specific immunoglobulin E is not sufficient for allergic nasal eosinophil infiltration in mice. Int Arch Allergy Immunol 161 Suppl 2: 125–128. 10.1159/000350565 23711863

[26] Nishimura T, Saeki M, Motoi Y, Kitamura N, Mori A, Kaminuma O, et al (2014) Selective suppression of Th2 cell-mediated lung eosinophilic inflammation by anti-major facilitator super family domain containing 10 monoclonal antibody. Allergol Int 63 Suppl 1: 29–35. 10.2332/allergolint.13-OA-0635 24809373

[27] Mori A, Ogawa K, Someya K, Kunori Y, Nagakubo D, Yoshie O, et al (2005) Selective suppression of Th2 cell-mediated airway eosinophilic inflammation by low-molecular weight CCR3 antagonist. Int Immunol 19: 913–921. 10.1093/intimm/dxm049 17804691

[28] Suzuki K, Kaminuma O, Yang L, Takai T, Mori A, Umezu-Goto M, et al (2011) Prevention of allergic asthma by vaccination with transgenic rice seed expressing mite allergen: induction of allergen-specific oral tolerance without bystander suppression. Plant Biotechnol J 9: 982–990. 10.1111/j.1467-7652.2011.00613.x 21447056

[29] Motoi Y, Saeki M, Nishimura T, Katayama K, Kitamura N, Ichikawa H, et al (2012) Establishment of monoclonal antibodies against a novel eosinophil-specific cell surface molecule, major facilitator super family domain containing 10. Immunol Lett 147: 80–84. 10.1016/j.imlet.2012.07.001 22820041

[30] Ponikau JU, Sherris DA, Kephart GM, Kern EB, Gaffey TA, Tarara JE, et al (2003) Features of airway remodeling and eosinophilic inflammation in chronic rhinosinusitis: is the histopathology similar to asthma? J Allergy Clin Immunol 112: 877–882. 14610473 10.1016/j.jaci.2003.08.009

[31] Katoh S, Kaminuma O, Hiroi T, Mori A, Ohtomo T, Maeda S, et al (2011) CD44 is critical for airway accumulation of antigen-specific Th2, but not Th1, cells induced by antigen challenge in mice. Eur J Immunol 41: 3198–3207. 10.1002/eji.201141521 21874648

[32] Di Lorenzo G, Mansueto P, Pacor ML, Martinelli N, Rizzo M, Ditta V, et al (2009) Clinical importance of eosinophil count in nasal fluid in patients with allergic and non-allergic rhinitis. Int J Immunopathol Pharmacol 22: 1077–1087. 20074472 10.1177/039463200902200424

[33] Siracusa MC, Kim BS, Spergel JM, Artis D (2013) Basophils and allergic inflammation. J Allergy Clin Immunol 132: 789–801; quiz 788. 10.1016/j.jaci.2013.07.046 24075190 PMC3903395

[34] Karasuyama H, Obata K, Wada T, Tsujimura Y, Mukai K (2011) Newly appreciated roles for basophils in allergy and protective immunity. Allergy 66: 1133–1141. 10.1111/j.1398-9995.2011.02613.x 21545430

[35] Long R, Zhou Y, Huang J, Peng L, Meng L, Zhu S, et al (2015) Bencycloquidium bromide inhibits nasal hypersecretion in a rat model of allergic rhinitis. Inflamm Res 64: 213–223. 10.1007/s00011-015-0800-6 25690567

[36] Wakasa Y, Takagi H, Hirose S, Yang L, Saeki M, Nishimura T, et al (2013) Oral immunotherapy with transgenic rice seed containing destructed Japanese cedar pollen allergens, Cry j 1 and Cry j 2, against Japanese cedar pollinosis. Plant Biotechnol J 11: 66–76. 10.1111/pbi.12007 23066780

[37] Saeki M, Nishimura T, Mori A, Kaminuma O, Hiroi T (2014) Antigen-induced mixed and separated inflammation in murine upper and lower airways. Allergol Int 63 Suppl 1: 59–61. 10.2332/allergolint.13-LE-0634 24809378

[38] Haenuki Y, Matsushita K, Futatsugi-Yumikura S, Ishii KJ, Kawagoe T, Imoto Y, et al (2012) A critical role of IL-33 in experimental allergic rhinitis. J Allergy Clin Immunol 130: 184–194e111. 10.1016/j.jaci.2012.02.013 22460070

[39] Saito H, Howie K, Wattie J, Denburg A, Ellis R, Inman MD, et al (2001) Allergen-induced murine upper airway inflammation: local and systemic changes in murine experimental allergic rhinitis. Immunology 104: 226–234. 11683963 10.1046/j.0019-2805.2001.01253.x PMC1783291

[40] Zhang Q, Lai K, Xie J, Chen G, Zhong N (2009) Does unrestrained single-chamber plethysmography provide a valid assessment of airway responsiveness in allergic BALB/c mice? Respir Res 10: 61 10.1186/1465-9921-10-61 19575792 PMC2719610

[41] Shin JH, Park HR, Kim SW, Park CS, Cho JH, Park YJ (2012) The effect of topical FK506 (tacrolimus) in a mouse model of allergic rhinitis. Am J Rhinol Allergy 26: e71–75. 10.2500/ajra.2012.26.3743 22487280

[42] Kato Y, Akasaki S, Muto-Haenuki Y, Fujieda S, Matsushita K, Yoshimoto T (2014) Nasal sensitization with ragweed pollen induces local-allergic-rhinitis-like symptoms in mice. PLOS ONE 9: e103540 10.1371/journal.pone.0103540 25119881 PMC4132107

[43] Bouley J, Groeme R, Le Mignon M, Jain K, Chabre H, Bordas-Le Floch V, et al (2015) Identification of the cysteine protease Amb a 11 as a novel major allergen from short ragweed. J Allergy Clin Immunol 136: 1055–1064. 10.1016/j.jaci.2015.03.001 25865353

[44] Sugahara S, Nabe T, Mizutani N, Takenaka H, Kohno S (2003) Kinins are involved in the development of allergic nasal hyperresponsiveness in guinea pigs. Eur J Pharmacol 476: 229–237. 12969770 10.1016/s0014-2999(03)02185-x

[45] Nabe T, Tsuzuike N, Ohtani Y, Mizutani N, Watanabe S, Fujii M, et al (2009) Important roles of tachykinins in the development of allergic nasal hyperresponsiveness in guinea-pigs. Clin Exp Allergy 39: 138–146. 10.1111/j.1365-2222.2008.03097.x 18778270

[46] Lai JP, Douglas SD, Ho WZ (1998) Human lymphocytes express substance P and its receptor. J Neuroimmunol 86: 80–86. 9655475 10.1016/s0165-5728(98)00025-3

[47] Blum A, Setiawan T, Hang L, Stoyanoff K, Weinstock JV (2008) Interleukin-12 (IL-12) and IL-23 induction of substance p synthesis in murine T cells and macrophages is subject to IL-10 and transforming growth factor beta regulation. Infect Immun 76: 3651–3656. 10.1128/IAI.00358-08 18505813 PMC2493212

[48] Cantalupo L, Cioni C, Annunziata P (2008) Expression of preprotachykinin-A mRNA isoforms and substance P production in T lymphocytes of human healthy subjects. Neurosci Lett 434: 191–194. 10.1016/j.neulet.2008.01.053 18294774

[49] Kraneveld AD, Folkerts G, Van Oosterhout AJ, Nijkamp FP (1997) Airway hyperresponsiveness: first eosinophils and then neuropeptides. Int J Immunopharmacol 19: 517–527. 9637348 10.1016/s0192-0561(97)00085-4

[50] Kay AB (2005) The role of eosinophils in the pathogenesis of asthma. Trends Mol Med 11: 148–152. 15823751 10.1016/j.molmed.2005.02.002

[51] Garcia G, Taille C, Laveneziana P, Bourdin A, Chanez P, Humbert M (2013) Anti-interleukin-5 therapy in severe asthma. Eur Respir Rev 22: 251–257. 10.1183/09059180.00004013 23997052 PMC9487362

[52] Koga H, Miyahara N, Fuchimoto Y, Ikeda G, Waseda K, Ono K, et al (2013) Inhibition of neutrophil elastase attenuates airway hyperresponsiveness and inflammation in a mouse model of secondary allergen challenge: neutrophil elastase inhibition attenuates allergic airway responses. Respir Res 14: 8 10.1186/1465-9921-14-8 23347423 PMC3570429

[53] Hughes JM, McKay KO, Johnson PR, Tragoulias S, Black JL, Armour CL (1993) Neutrophil-induced human bronchial hyperresponsiveness in vitro—pharmacological modulation. Clin Exp Allergy 23: 251–256. 8391372 10.1111/j.1365-2222.1993.tb00318.x

