# Supplementary figures and images for: Essential Contribution of CD4+ T Cells to Antigen-Induced Nasal Hyperresponsiveness in Experimental Allergic Rhinitis
Source: PLoS One. 2016 Jan 11;11(1):e0146686. doi: 10.1371/journal.pone.0146686 (PMC4709066; doi:10.1371/journal.pone.0146686)

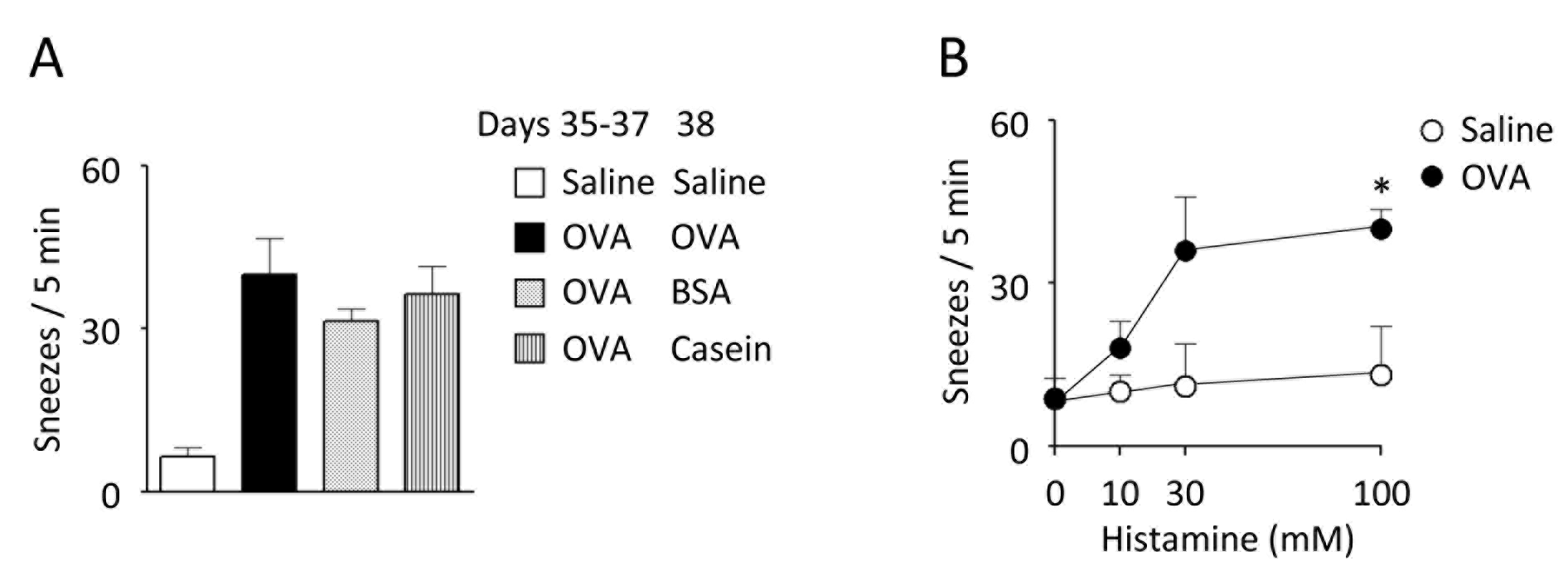

Supplement: S1 Fig — The number of sneezes evoked by OVA, BSA, or casein (A), or several concentrations of histamine (B) in OVA-immunized and saline- or OVA-challenged mice (N = 4–8). (TIF) [file pone.0146686.s001.tif]

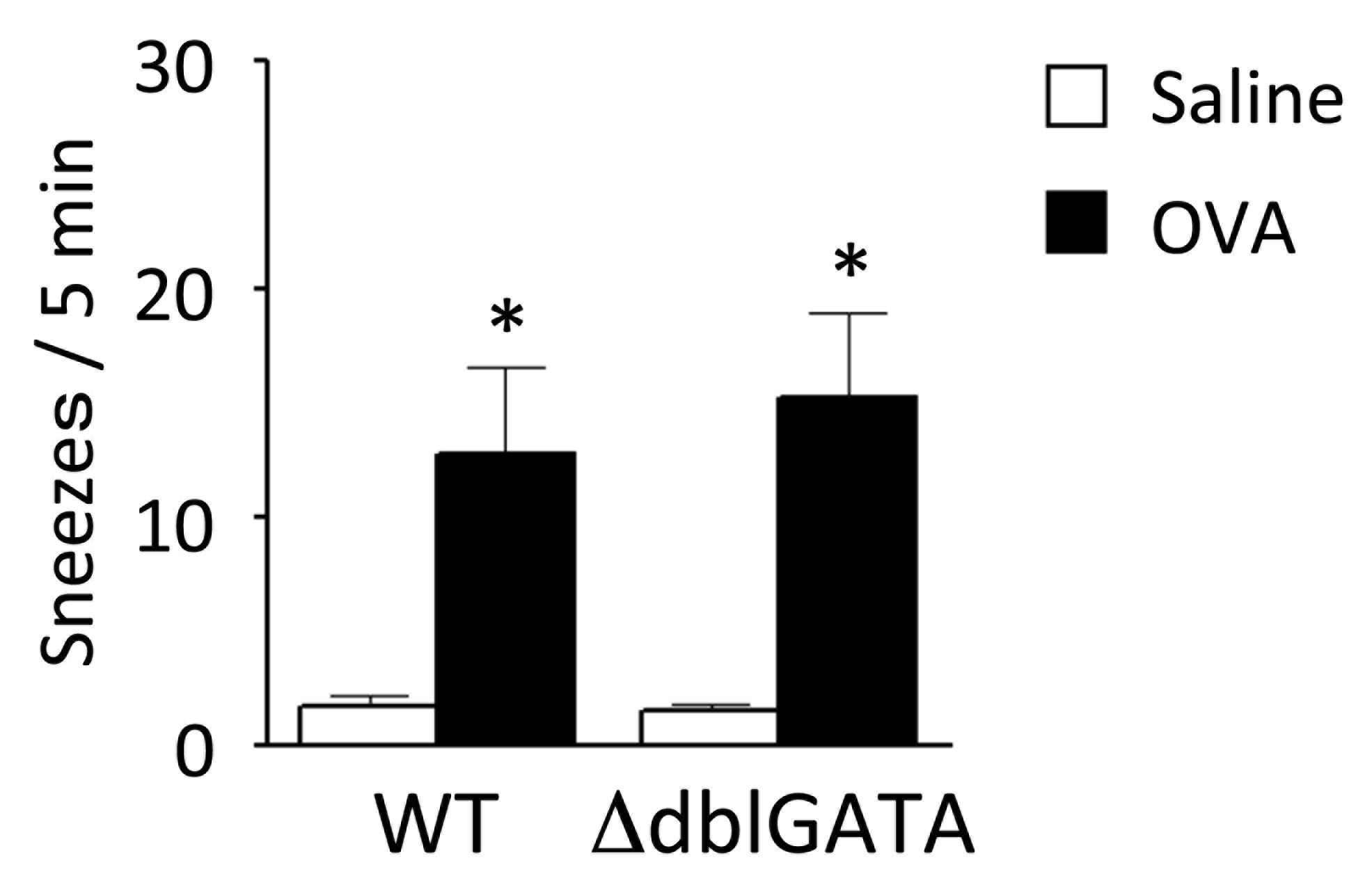

Supplement: S2 Fig — The number of sneezes evoked by BSA in OVA-immunized and saline- or OVA-challenged ΔdblGATA and WT mice (N = 4–10). (TIF) [file pone.0146686.s002.tif]
